# Molecular characteristics of glutathione transferase gene family in a neglect medical *Spirometra* tapeworm

**DOI:** 10.3389/fvets.2022.1035767

**Published:** 2022-11-02

**Authors:** Wen Qing Chen, Sha Sha Liu, Chi Cheng, Jing Cui, Zhong Quan Wang, Xi Zhang

**Affiliations:** Department of Parasitology, School of Basic Medical Sciences, Zhengzhou University, Zhengzhou, China

**Keywords:** cestode, glutathione transferase, molecular characterization, enzymatic traits, phylogeny

## Abstract

The *Spirometra mansoni* is a neglect medical tapeworm, its plerocercoid larvae can parasitize in humans and animals, causing sparganosis. In this study, 17 new members of the glutathione transferase (GST) family were sequenced and characterized in *S. mansoni*. Clustering analysis displayed the categorization of SmGSTs into two main clades. RT-qPCR illustrated that 7 GST genes were highly expressed in the plerocercoid stage while 8 GSTs were highly expressed in the adult. rSmGST has the typical C- and N-terminal double domains of glutathione transferase. Immunolocalization revealed that natural SmGST is mainly located in the epidermis and parenchyma of plerocercoid, and in the epidermis, parenchyma, uterus and egg shell of adult worm. The optimum activity for rSmGST was found to be pH 6.5 and 25°C. The evolutionary tree showed a high level of diversity of cestodes GSTs. SmGSTs contained both conserved family members and members in the process of further diversification. The findings in this study will lay a foundation to better explore the underlying mechanisms of GSTs involved in *Spirometra* tapeworms.

## Introduction

The plerocercoid larvae of *Spirometra* (Cestoda: Diphyllobothriidae) tapeworms can parasitize in humans and animals, causing a food/water-borne parasitic zoonosis known as sparganosis (1). Sparganosis typically manifests as migrating larvae, and the symptoms depend on their localization in the body. In humans, the plerocercoid can invade the subcutaneous tissues, spinal cord, eyes, breasts and brain, resulting in local tissue damage, paralysis, blindness, and even death (2). Until now, more than 2,000 human sparganosis have been reported, with the majority originating from east and southeast Asian countries (3). Recently, plerocercoid infections in humans and animals have also been reported in Africa, America and Europe (4). Although several methods have been introduced in diagnosis and treatment, precision medicine for sparganosis still has a long way to go (5). Understanding the intricacies of the parasite and its interactions with hosts is central to developing new intervention strategies (6). Several parasite molecules have been implicated in the interference with the host response, such as the glutathione transferases (GSTs), which is a super family of enzymes found ubiquitously in organisms (7–10).

GSTs detoxify endogenous and xenobiotic electrophilic toxins by catalyzing their conjugation with glutathione (GSH). Hence, many parasitic helminth GSTs are considered to be important targets for the treatment or immune intervention of parasitic infections (11). Current studies have confirmed that glutathione transferase displayed effective protective immunity in experimental animals for antihelminth (12, 13). According to their amino acid conservation and phylogenetic inferences, the GST superfamily can be subdivided into four independent and unrelated families: cytosolic GSTs (cGSTs), microsomal GSTs (MAPEG), mitochondrial GSTs (kappa GSTs or GSTK) and bacterial GSTs (14). Among them, cGSTs is the major group with members in all aerobic organisms, and can be further divided into 7 classes: Alpha, Mu, Omega, Pi, Sigma, Theta, and Zeta in mammals (7, 15). With the completion of the helminth genome projects, a large number of genomic datasets across a broad range of cestodes were deposited in WormBase ParaSite database. On the basis of genome and transcriptome data libraries, numerous GST sequences have been isolated and recognized in multiple cestodes (16–18). Currently, MAPEG and types of Mu, Omega and Sigma cGSTs have been identified in cestodes (7, 16). However, the GST genes have never been studied in *Spirometra* species, our knowledge about the family structures and molecular features of GSTs in this medical tapeworm is still fragmentary.

Nowadays, the first draft genome of *S. mansoni* was sequenced (19), and the expressed sequence tags (ESTs) were functionally analyzed (20). Recently, a phosphoproteomic analysis and a transcriptomic analysis of *S. mansoni* were performed (6, 21). These publicly available datasets offer opportunities to perform detailed analysis of the GST family in *Spirometra* tapeworms. More importantly, previous genomic and transcriptomic analysis showed highly expressed of GST genes in *S. mansoni*, indicating important roles of GST in the life cycle of *Spirometra* tapeworms (6, 19). Therefore, understanding the structure, molecular characteristics and evolutionary pattern of the GST family will be helpful for understanding the interactions between parasite and host, as well as developing new intervention strategies for sparganosis. Specifically, the aims of this study are as follows: (1) offers a detailed analysis of the GST family in *S. mansoni* and investigates the molecular characterization; and (2) builds a framework for the evolution of the GST family in the cestodes to explain the sequence and functional diversity of this gene family.

## Materials and methods

### Ethical approval

This study was approved by the Life Science Ethics Committee of Zhengzhou University (Permit code. SYXK 2020-1127). The animals were handled in accordance with good animal practices required by the Animal Ethics Procedures and Guidelines of the People's Republic of China.

### Experimental animals

The plerocercoid of *Spirometra* tapeworms were isolated from wild frogs (*Pelophylax nigromaculatus*) using method described before (22). The collected worms were tentatively identified as *S. mansoni* by molecular typing based on the mitochondrial cytochrome *c* oxidase subunit 1 (*cox*1) gene according to the method detailed in Kuchta et al. (3). Collected plerocercoids were orally administered to female specific pathogen-free (SPF) mice (3 plerocercoids per mouse) and maintained by serial passage in mice every 10–12 months. Positive serum against plerocercoid was obtained from the conserved mice. Twenty female BALB/c mice (4~6-week-old) were used to immunize by antigens of recombinant glutathione transferase (rGST) to obtain the anti-rSmGST serum. The rSmGST was produced in *Escherichia coli* and purified with Ni^2+^ affinity chromatography. Twenty BALBc mice were immunized for 4 times. All anti-rSmGST serum was stored at −80°C until used. An adult cestode representing *S. mansoni* was obtained from an infected domestic cat as described previously (1). Proglottids collected from the adult were used for the subsequent experiments.

### Identification of GST family members

Genes encoding proteins that contain the glutathione transferase domain in the *S. mansoni* were searched using the NCBI conserved domains database. All candidate SmGSTs were obtained from the WormBase ParaSite database and the transcriptomic data (6). These extracted sequences were identified belong to the GST family by querying for genes annotated with the Pfam domain accessions PF02798, PF14497 and PF00043 for cytosolic GSTs, and PF01124 for MAPEG. All identified candidates were analyzed using the HMMER tool to confirm the presence of GST_N, GST_C or MAPEG domains in their protein structure (23). For candidate genes from the transcriptomic data, the nucleotide sequences were firstly translated to amino acid sequences using the NCBI's ORF finder tool and using BLASTX for homology searches. Finally, these retrieved sequences were corroborated by cloning and sequencing of *S. mansoni* cDNAs. All obtained SmGST sequences were deposited in GeneBank under accession numbers ON527155 to ON527171. The molecular weights, theoretical pI values, and number of amino acids for the identified SmGSTs were predicted using the ExPASy (https://www.expasy.org). The subcelluar localization was predicted by TargetP (www.cbs.dtu.dk/services/TargetP). The conserved protein motif analysis was performed using the mixture model by expectation maximization (MEME) method. Motif scan and NCBI-CDD server (https://www.ncbi.nlm.nih.gov/Structure/cdd) were used for conserved functional protein domain prediction. The phylogenetic tree was inferred using maximum likelihood (ML) method based on the LG + G model. The ML analysis was performed in MEGA v7 with 1,000 bootstrap replications (24). The three-dimensional structure was determined using homology modeling available at the SwissModel server. The quality of the model was examined using Ramachandran plot analysis and visualized by the Swiss-PdbViewer v.4.1 (25).

### Quantitative RT-PCR analysis

Quantitative RT-PCR (qRT-PCR) analysis was performed to monitor the expression levels of identified SmGSTs in two life cycle stages of *S. mansoni*: plerocercoid stage and adult stage (including immature proglottide, mature proglottide and gravid proglottide). The gene-specific primers are listed in Supplementary Table S1. Total RNA was isolated using a reverse transcription kit (Novoprotein, Shanghai, China). qRT-PCR was conducted on a 7,500 Fast Real-time PCR system (Applied Biosystem, Monza, Italy). The reaction mixture contained 10 μL of 2 × TB Green Premix Ex Taq (Takara, Beijing, China), 10 μM each of sense and antisense primers, 100 ng of first-strand cDNA. Initial thermal-cycling at 95°C for 30 s followed by 40 cycles of 95°C for 3 s and 60°C for 30 s. The GAPDH gene was served as the internal control (26). Relative gene expression levels were analyzed according to the comparative 2^−Δ*ΔCT*^ method (27).

### Cloning, expression and identification of rSmGST

The first *Spirometra* GST gene deposited in GeneBank (AEI16476.1) was amplified by PCR with specific primers carrying BamHI and PstI restriction enzyme sites (underlined) (forward, 5′-ATGGATCCATGGGTTC GCTCCCGGTTC-3′, and reverse, 5′-ATCTGCAGCTAAGCATCACCACGCCAG-3′), and the cycling protocol was as follows: 30 cycles of 95°C for 50 s, 60°C for 50 s and 72°C for 50 s. The final PCR products were purified, digested, and cloned into the pQE-80L vector (Ipswich, USA). The recombinant plasmid was then transformed into *Escherichia coli* BL21 (New England Biolabs, USA). Expression of rSmGST was induced by adding 0.5 mM IPTG at 37°C for 4 h. The rSmGST was purified by Ni^2+^ affinity chromatography (Shenggong Biotech, Shanghai, China) and identified by SDS-PAGE. Images of gels were recorded using ImageScanner (GE Healthcare, Fairfield, CT). Another gel was prepared by the same method and used for the western blotting analysis.

### Development of indirect ELISA

Antibody titrations of immunized mice were performed by indirect ELISA. The rSmGST protein was coated onto each well of 96-well plates (BIOFIL, Guangzhou, China) overnight at 4°C. Protein-coated plates were blocked with 5% skim milk at 37°C for 2 h. All primary antibodies were diluted with PBS-0.05% Tween 20 (PBST), and added onto precoated ELISA plates with incubation at 37°C for 2 h. HRP-labeled goat anti-mouse IgG (EarthOX, USA) was added at 1: 5,000 dilution and incubated for 1 h at 37°C. Finally, 100 μL OPD chromogen substrate containing H_2_O_2_ were added to each well and the plates incubated, protected from the light, for 15 min and the reaction was stopped by the addition of 50 μL 2 M H_2_SO_4_. The optical density (OD) of all the wells was measured at 490 nm using a computer-controlled BioTek (Synergy LX, USA) microplate reader.

### Indirect immunofluorescence assay

IFA was used to locate the position of SmGSTs. The tissue sections of plerocercoids and adult worms was first retrieved after microwaving for 20 min with a 0.01 M citric acid buffer (pH 6.0), blocking with 5% normal goat serum in PBS, then incubating at 37°C or 1 h with a 1: 10 dilution of anti-rSmGST serum, serum of mice infected with plerocercoids, and normal mouse serum or PBS, respectively. The sections were incubated with a 1: 50 dilution of FITC-labeled anti-mouse IgG (Santa Cruz, USA), and the nuclei were stained with propidium iodide (PI) at 37°C for 15 min. Finally, the sections were examined under a fluorescent microscope (Olympus, Japan) after washing five times with PBS.

### Enzyme activity assays

The effect of temperature on rSmGST was carried out by keeping the enzyme at different temperatures (15, 20, 25, 30, 35, 40, 45, 50, and 55°C) for 10 min, and then enzyme activity was assayed immediately. The effect of pH on rSmGST was carried out using the phosphate buffer having different pH from 5.5 to 10. Activity of enzyme was determined in 100 mM potassium phosphate buffer (pH 6.5) with 1 mM l-chloro-2,4-dinitrobenzene (CDNB) and 4 mM GSH as substrates by following the change in absorbance at 340 nm (ε $\frac{1}{4}$ 9.6 mM^−1^. cm^−1^) for 1 min at 30°C as previously described (28). In the kinetic mechanism analysis of rSmGST, double reciprocal Lineweaver-Burk plot was used to graphically determine the apparent *K*_*m*_ and *V*_*max*_ values. Inhibition studies were performed according to the procedure of Tahir et al. (29). Different range of concentrations of Rose Bengal (RB, 0.1–1000 μM), Cibacron Blue (CB, 0.1–1000 μM), Bromosulfophtalein (BSP, 0.1–500 μM), and Tripheniltin chloride (TPT, 0.1–100 μM) were used as inhibitors respectively. The concentration of inhibitor giving 50% inhibition (IC_50_) was obtained by plotting the percent of residual activity *vs* the log of inhibitor concentration. The *K*_*i*_ values for each rSmGST inhibitor were determined according to methods described in Nava et al. (30). To ascertain which type of reversible enzyme inhibition occurs, initial-velocity enzyme reactions in the presence of the inhibitors were performed. The rSmGST was preincubated for a few seconds in the assay buffer with 4 mM GSH and inhibitor at the corresponding concentration (CB, 10, 30 μM; TPT, 4, 8 μM; BSP, 20, 60 μM and RB, 20, 60 μM). The reaction was initiated by addition of one of a range of 0.20 to 2.0 mM of different concentrations of CDNB. A set of reactions under identical conditions was performed for each inhibitor concentration and for the non-inhibitor control.

### Phylogenetic analysis

The GST sequences of 15 other medical cestodes (*Taenia solium, Taenia saginata, Taenia asiatica, Taenia multiceps, Echinococcus granulosus, Echinococcus multilocularis, Echinococcus canadensis, Hymenolepis microstoma, Hymenolepis nana, Hymenolepis diminuta, Hydatigera taeniaeformis, Mesocestoides corti, Acanthocheilonema viteae, Schistocephalus solidus* and *Dibothriocephalus latus*) were also extracted from the WormBase ParaSite database. GSTs motif analysis was performed using the mixture model by expectation maximization (MEME) method (31). The motif search is performed using the default parameters, has a maximum width of 50 amino acids, and allows the motif in the sequence to be repeated any number of times. Multiple sequence alignment was performed using MAFFT v7 (32). Phylogenetic analyses were performed using two methods of Bayesian inference (BI) and Maximum likelihood (ML) respectively. Protein sequences were aligned with MAFFT v7 using the FFT-NS-I method. The best substitution model was defined with the Smart Model Selection (SMS) tool (33) incorporated in PhyML v3.0 (34). The ML tree was generated with PhyML using the aLRT-SH method for branch support. The BI tree was generated with BEAST v1.8.4 (35) using two independent runs of 50,000,000 chains and sampling at every 5,000 generations. The software TRACER v1.6 was used to check the convergence of Monte Carlo Markov Chains (MCMC) and to ensure adequate effective sample sizes (ESS > 200) after the first 10% of generations were deleted as burn-in. The maximum clade credibility tree was estimated with TreeAnnotator, which is part of the BEAST v1.8.4 package, and the tree was visualized using Figtree v1.4.

## Results

### Manual annotation of SmGST genes

A total of 17 GST members were identified in *S. mansoni*, 8 sequences (ON527155-ON527162) were originally screened from the WormBase ParaSite database, the remaining 9 sequences (ON527163-ON527171) were identified from the transcriptomic data (Table 1). Among the 17 SmGSTs, 15 were identified as cGSTs and 2 belong to MAPEG (ON527159 and ON527167). Within 15 cGSTs, 10 Mu types (ON527155-ON527157, ON527160, ON527161, ON527164, and ON527168-ON527171) and 2 Sigma types (ON527158 and ON527165) were classified. The length of SmGSTs ranged from 216 bp to 672 bp. The predicted protein length ranged from 72 aa to 224 aa. In case of domain length, the length ranged from 25 aa to 156 aa. The molecular weights varied from 7.8 kDa to 25.3 kDa, while the theoretical isoelectric points ranged from 4.40 to 10.02.

**Table 1:** Annotations features for glutathione transferase of *Spirometra mansoni*.

**Gene ID**	**CDS length (bp)**	**Protein length (aa)**	**GST domain coordinates**	**Domain length (aa)**	**Mw (Da)**	**PI**	**Subcellular location**
ON527155	252	84	1–73	73	9,867.29	5.70	Other
ON527156	489	163	2–58, 68–163	57, 96	18,842.48	4.98	Other
ON527157	447	149	6–44, 54–149	39, 96	17,278.90	5.93	Other
ON527158	270	89	1–69	69	10,089.89	9.77	Other
ON527159	351	117	1–103	103	13,193.70	10.02	Mitochondrion
ON527160	216	72	1–25	25	7,808.04	6.93	Other
ON527161	585	195	1–56, 65–183	56, 119	22,265.25	5.20	Other
ON527162	492	164	76–136	61	18,310.84	4.40	Other
ON527163	615	205	1–71, 85–186	71, 102	23,550.62	7.07	Secretory pathway
ON527164	615	205	1–67, 75–195	67, 121	23,427.80	6.03	Other
ON527165	579	193	2–70, 10–146	69, 137	22,034.72	9.20	Other
ON527166	672	224	3–158, 127–200	156, 74	25,352.81	4.62	Other
ON527167	351	117	1–103	103	13,227.72	10.02	Mitochondrion
ON527168	621	207	4–72, 80–198	69, 119	24,153.40	5.56	Other
ON527169	330	110	1–99	99	12,924.72	4.98	Other
ON527170	531	177	4–38, 59–166	35, 108	20,190.15	5.33	Other
ON527171	543	181	1–74, 83–181	74, 99	20,937.89	4.71	Other

**Figure 1:**
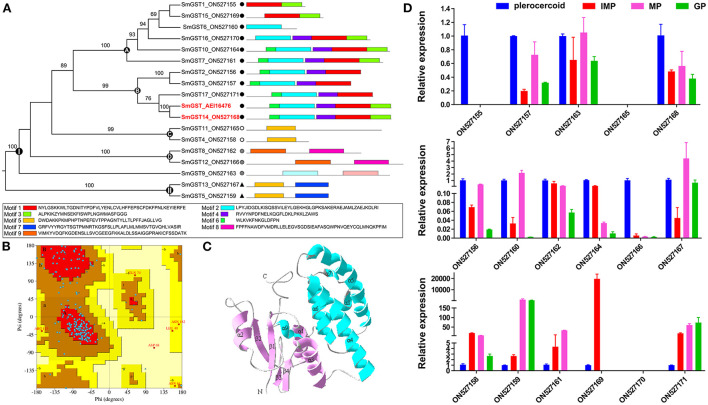
Glutathione transferase protein family members identified in *Spirometra mansoni*. **(A)** Phylogenetic tree and conserved motifs of SmGSTs. The symbol “▴” indicates microsomal GSTs (MAPEG), “•” indicates Mu class cGST, “°” indicates Sigma class cGST, “⊗” indicates unknown class cGST. **(B)** Model quality evaluation of 3D structure of SmGST. Red represents the most favored regions, brown indicates additional allowed regions, yellow indicates the generously allowed regions, light color indicates disallowed regions. **(C)** Ribbon protein structures of SmGST. Swiss-model protein modeling server was used for modeling; α-helices show as ribbons and β-strands as arrows. Pink: the thioredoxin-like domain (N-terminal GSH binding domain), Green: C-terminal substrate binding domain. **(D)** GST genes expression of *S. mansoni* in different tissues by qRT-PCR. The expression level was normalized to GAPDH and measured with 2^−Δ*ΔCt*^ value. Results are averaged from three independent replicates during all stages. Error bars represent SD (*n* = 3). IMP, immature proglottide; MP, mature proglottide; GP, gravid proglottide.

A phylogenetic analysis based on full-length protein sequences revealed that the SmGSTs can be arranged into 2 main clades: Clade I and Clade II (Figure 1A). Clade I included all cGSTs and clade II contained the remaining 2 MAPEG GSTs. Within the clade I, 4 robust supported groups were revealed: groups A-D. GST members in group A and group B can be further identified as Mu class GST, and GSTs in group C were identified as Sigma class. The MEME program determined 9 specific putative motifs that contain 14 to 50 residues (Figure 1A; Supplementary Table S2). The motif patterns within each group have similar organizations. The 3D homology analysis of the selected sequence (AEI16476.1) showed that GST protein containing two soluble domains: a Mu class N-terminal domain (residues 5-86), and a C-terminal domain (94-212). In detail, 8 G-sites were identified in the N-terminal at the positions of Y9, W10, W48, K52, N61, L62, Q74, and Q75, respectively; and 5 H-sites were observed in the C-terminal at S107, R110, A111, F165, and A168. The motif scan analysis revealed that SmGST consists of a casein kinase II phosphorylation site (92–95) and a protein kinase C phosphorylation site (92–94). The Ramachandran plot analysis showed that 93.1% of the residues (352 aa) located in favored region, 5.8% of the residues (22 aa) located in allowed region and only 1.1% of the residues (4 aa) located in outlier region, suggesting high quality of the protein model (Figure 1B). Structural homology model showed the formation of the N-terminal domain and the C-terminal domain in *S. mansoni* GST (Figure 1C). The N-terminal domain contained β sheets with a β_1_α_1_β_2_α_2_β_3_β_4_α_3_ thioredoxin fold while the C-terminal domain had an entirely helical structure. The two domains were joined by a linker with a coil structure. In addition, a special mu loop (EAGGPPDFS) between β_2_ and α_2_ was found.

To profile the expression patterns of identified SmGSTs, we sampled plerocercoid and different proglottides of adults for analysis by qRT-PCR (Figure 1D). A total of 15 SmGSTs were detected with expression in both plerocercoid and adult stages. Among these 15 SmGSTs, 7 genes were highly expressed in the plerocercoid stage, while 8 were highly expressed in the adult stage. In the adult stage, 14 genes were detected. Among which, one gene (ON527161) was expressed both in the immature proglottide and mature proglottide, ON527169 was specifically expressed in the immature proglottide and ON527155 was expressed only in the plerocercoid stage.

### Expression of rSmGST

**Figure 2:**
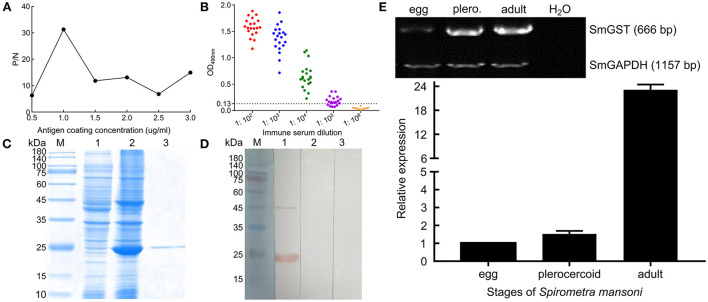
Molecular characterization of cloned SmGST. **(A)** Determination of optimal antigen coating concentration. **(B)** Determination of anti-rSmGST immune serum titer by indirect ELISA. Red, blue, green, purple, and orange represent serum dilutions of 1:10^2^, 1:10^3^, 1:10^4^, 1:10^5^, and 1:10^6^, respectively. **(C)** SDS-PAGE analysis of 10 μg purified GST from *S. mansoni* on 12% gel. M: protein pre-staining marker; Lane 1: uninduced bacterial cultures; Lane 2: the lysate of the induced recombinant bacteria harboring pQE-80L-rSmGST after ultrasonication; Lane 3: rSmGST purified by Ni-NTA-Sefinose Column. **(D)** rSmGST antigenicity analysis. M: protein pre-staining marker; Lane 1: rSmGST + anti-rSmGST serum; Lane 2: rSmGST + infected mouse serum; Lane 3: rSmGST + pre-immune serum. **(E)** The transcription pattern of GST gene in different developmental stages of *Spirometra mansoni*. Conventional RT-PCR (upper) and real-time RT-PCR (lower) were performed on cDNA from various developmental stages of *S. mansoni*, including eggs, plerocercoid and adult. A house keeping gene (Se-GAPDH) was used as a positive control. H_2_O was used as a negative control.

**Figure 3:**
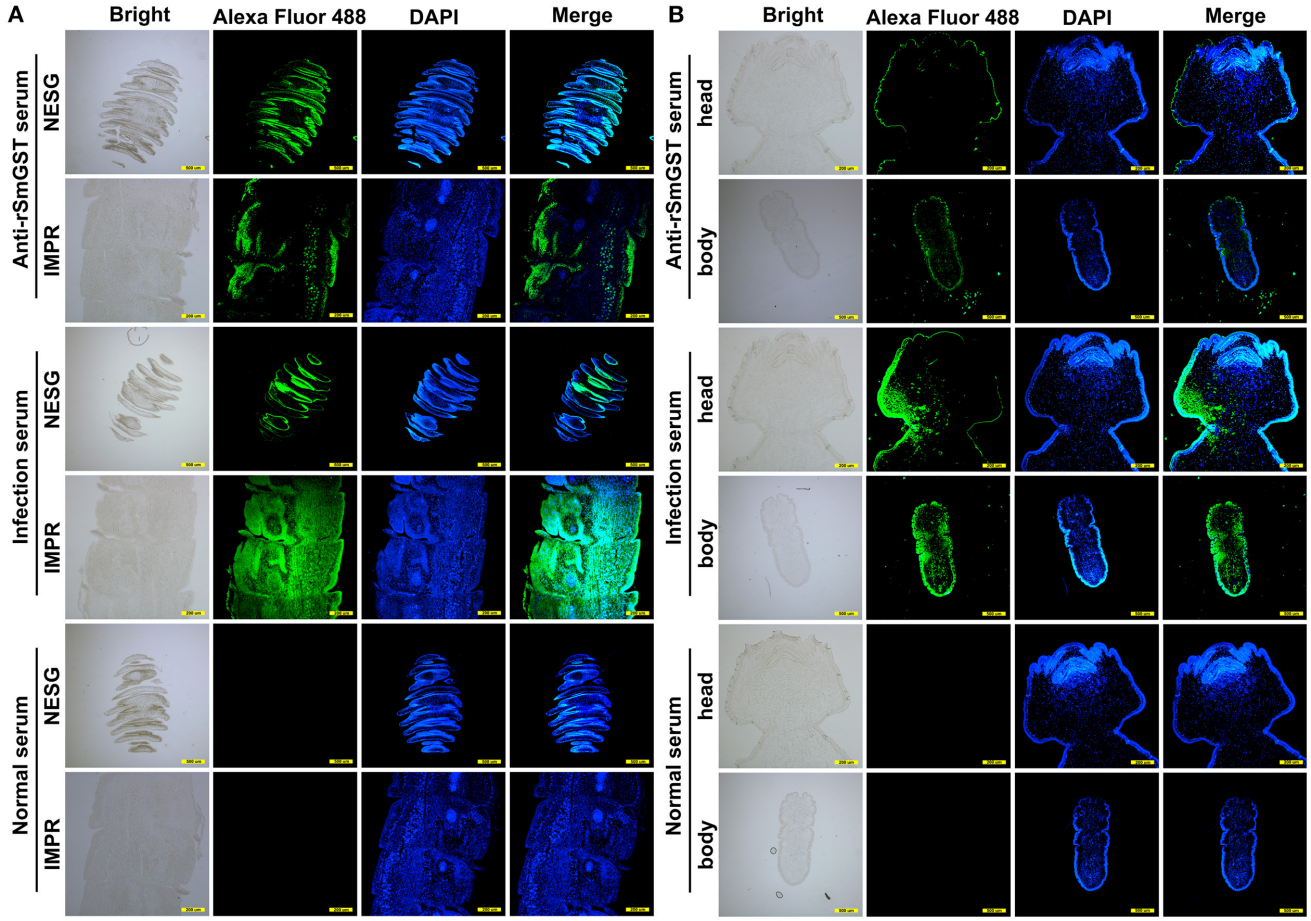
Immunofluorescence localization of GST in different developmental stages of *Spirometra mansoni*. **(A)** GST location in different segments in the adult stage. IMPR indicates immature proglottide, NESG indicates neck segment. **(B)** GST location in the plerocercoid stage. Green fluorescence is the location of GST protein. Scale of different segments of adult: 500 μm; Head of plerocercoid: 200 μm; Body of plerocercoid: 500 μm.

The molecular biological analysis showed that SmGST is a soluble protein with a predicted Mw of 25.8 kDa and a pI of 5.98, containing 7 serine-specific sites, 3 threonine- specific sites and 4 tyrosine-specific phosphorylation sites (Supplementary Figure S1). Molecular docking showed two hydrogen bonds between GSH and Arg110 residue, and between GSH and Ala18 residue. A hydrogen bond was formed between CDNB and Ala18 residue (Supplementary Figure S2). The sequence of SmGST cloned in this study was 99% identical to the reference gene (AEI16476.1). The coding sequence of the SmGST gene was cloned into the prokaryotic expression vector pQE-80L, and BL21 bacteria harboring the recombinant plasmid pQE-80L-SmGST expressed a soluble fusion protein. Additionally, we established an indirect ELISA using the rSmGST. A protein concentration of 1.0 μg/mL and a mouse sera dilution of 1: 100 were the optimal conditions (Figure 2A). And the cut-off value of 0.13 was used as a standard for the subsequent tests (Figure 2B). On SDS-PAGE analysis, the molecular size of rSmGST was 28 kDa and consistent with the predicted molecular size (Figure 2C). The concentration of rSmGST was 2.5 mg/ml. Western blotting analysis showed that rSmGST was recognized by the anti-rSmGST serum but unrecognized by the serum of infected mice with plerocercoids (Figure 2D). The mRNA transcription (666 bp) for the SmGST gene was observed at stages of egg, plerocercoid and adult worm. The qPCR analysis showed that the transcriptional level of the adult stage was the highest, followed by the egg stage and the plerocercoid stage (Figure 2E). The immunolocalization showed that specific fluorescent staining was observed in epidermis, parenchymas and uterus of adult worms using anti-rSmGST serum, and significant fluorescence was also observed in eggshells. In the plerocercoid stage, fluorescent staining was detected in subcutaneous and some parenchymal tissues (Figure 3; Supplementary Figure S3).

### Enzyme kinetics and inhibition studies

**Figure 4:**
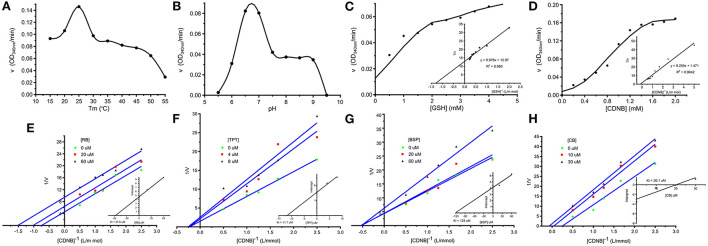
Enzymatic characteristics of SmGST. **(A)** Effects of temperature on rSmGST enzymatic activity. **(B)** Effects of pH on rSmGST enzymatic activity. **(C)** Effects of substrate concentration of GSH on rSmGST enzymatic activity. The kinetic parameters, *K*_*m*_ and *V*_*max*_, were determined using the Lineweaver–Burk's plot. The *K*_*m*_ and *V*_*max*_ values were 0.78 mM and 1.146 μmol min^−1^ mL^−1^, respectively. **(D)** Effects of substrate concentration of CDNB on rSmGST enzymatic activity. The *K*_*m*_ and *V*_*max*_ values were 6.29 mM and 10.120 μmol min^−1^ mL^−1^, respectively. **(E)** The effect of different concentrations of Rose Bengal (RB) on the initial velocities. **(F)** The effect of different concentrations of Tripheniltin chloride (TPT) on the initial velocities. **(G)** The effect of different concentrations of Bromosulfophtalein (BSP) on the initial velocities. **(H)** The effect of different concentrations of Cibacron Blue (CB) on the initial velocities. Inset shows secondary plot of the 1/*V*_*max*_ values derived from the primary Lineweaver–Burk plot vs. concentration for the determination of *K*_*i*_. **(E)** RB (20, 60 μM); **(F)** TPT (4, 8 μM); **(G)** BSP (20, 60 μM); **(H)** CB (10, 30 μM).

The affinity-purified rSmGST showed parabolic change in enzyme activity when the enzyme was incubated at temperatures between 15 and 30°C, and the optimum temperature for enzyme activity was 25°C (Figure 4A). The enzyme activity varied with change in pH, and the maximum GST activity was observed at pH 6.5 (Figure 4B). Kinetic study with rSmGST showed Michaelis–Menten behavior for GST with respect to the substrates GSH and CDNB. It was observed that by increasing the GSH concentration (0.5–5.0 mM), the activity of SmGST was slightly increased (Figure 4C). Then, it increased to certain level and reached the saturation point at 4.0 mM of GSH. A further increase in enzyme activity was not observed by increasing the concentration of the substrate. A similar trend was observed with increase in CDNB concentration (0.2–2.0 mM) showing a saturation point at 1.6 mM (Figure 4D).

In the inhibition studies, IC_50_ results for the SmGST were dependent on the inhibitor used. TPT was the most powerful inhibitor, with an IC_50_ of 12.1 μM. The corresponding IC_50_ values for CB, BSP, and RB were 11.66, 289.5, and 33.95 μM, respectively (Table 2). Plotting of the 1/v (μM min^−1^) axis intercept against inhibitor concentration yielded a *K*_*i*_ value of 57.6 μM for RB. The same procedure yielded *K*_*i*_ values of 11.7 μM for TPT, 122 μM for BSP, and 20.1 μM for CB (Figures 4E–H). An uncompetitive inhibition pattern was observed for RB and CB as inhibitors when CDNB was used as the variable substrate. And a non-competitive inhibition pattern was observed for the inhibitors BSP and TPT. Supplementary Figure S4 showed the plot composition of FV for SmGST vs. the logarithm of inhibitor concentrations with RB, BSP, TPT, and CB. Each straight line for a specific inhibitor indicated the maximal slope expected at IC_50_. The corresponding values were −0.27, −0.59, −0.63, and −0.51, respectively, for BSP, TPT, RB, and CB.

**Table 2:** Inhibition of SmGST by various classic glutathione transferase inhibitors.

**Inhibitor**	**IC_50_ (μM)**	**K_i_ (μM)**
Rose Bengal	33.95	57.6
Tripheniltin chloride	12.1	11.7
Cibacron Blue	11.66	20.1
Bromosulfophtalein	289.5	122

### GSTs in cestodes

**Figure 5:**
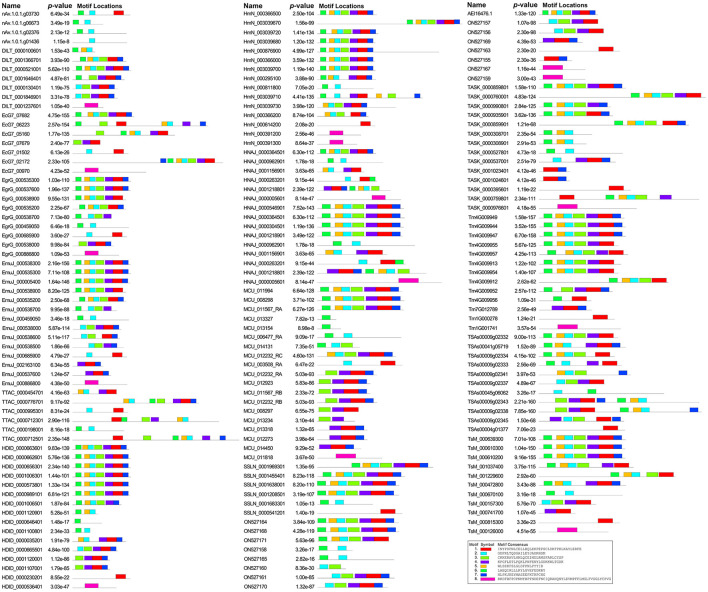
Conserved motifs of GST sequence motifs in medical cestodes.

A total of 182 GST sequences in 16 cestode species were retrieved from the public databases (Supplementary Table S3). The number of GST genes varied between cestode species: 20 genes in *M. corti*, 18 in *S. mansoni*, 16 in *H. diminuta*, 15 in *H. microstoma*, 14 in *E. multilocularis* and *T. asiatica*, 13 in *T. multiceps*, 12 in *T. saginata*, 11 in *T. solium*, 9 in *E. granulosus* and *H. nana*, 7 in *D. latus* and *E. canadensis*, 6 in *S. solidus* and *H. taeniaeformis*, and *A. viteae* only consisted of 5 GST members. The MEME program determined 8 highly conserved specific putative motifs and a total of 49 motif permutations were found (Figure 5). The motif permutation of 6 + 5 + 2 + 3 + 4 + 1 + 7 was the most frequently appeared, and it was widely distributed in all species except *A. viteae, T. asiatica, H. taeniaeformis* and *T. saginata*. Followed by motif 6 + 2, motif 8, motif 6 + 5 + 2 + 3 + 4 + 1 and motif 1. In addition, 25 special motif permutations were identified, such as motif 6 + 2 + 1, motif 3 + 6 + 5 + 2 + 4 +1 and motif 7 + 6 + 5 + 2 + 3 + 1. In addition, 4 single motifs (motif 1, 2, 6 and 8) were found in 28 sequences.

**Figure 6:**
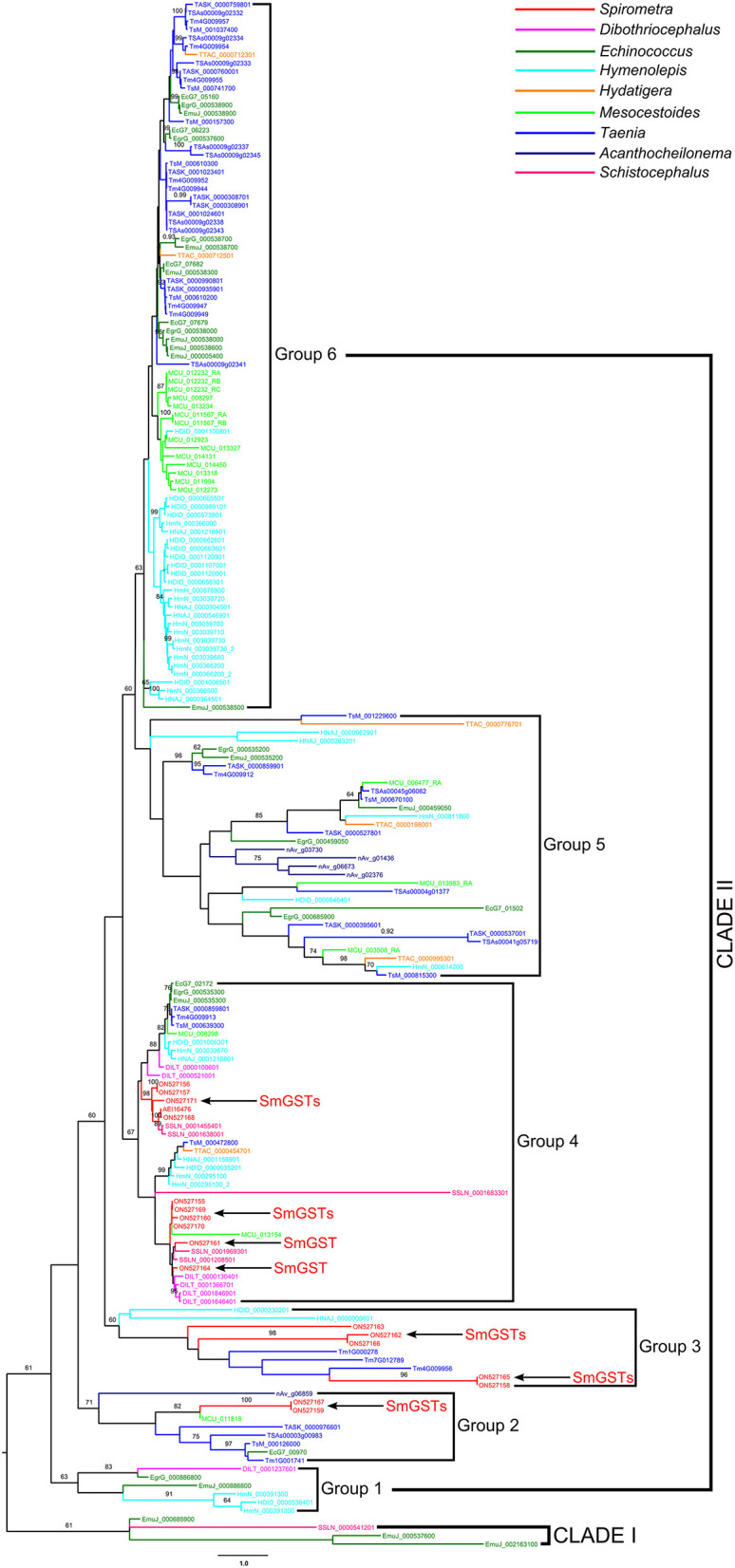
Phylogenetic analysis of glutathione transferase sequences in selected medical cestodes based on maximum likelihood method. The numbers on the branches represent bootstrap values, and only values with bootstrap values >60 are displayed.

The model tests suggested that the JTT + G model was the most suitable for GST alignments. In the phylogenetic analysis, both the maximum likelihood and Bayesian methods generated consistent tree topologies (Figure 6). The tree topology suggested that all GSTs can be divided into two branches: Clade I and Clade II. Clade I contained 3 sequences of *E. multilocularis* and a sequence of *S. solidus*. In contrast, the Clade II including a larger number of sequences and can be further divided into six groups: Group 1 to Group 6. Within Clade II, the earliest diversification firstly gave rise to the Group 1, then to Group 2, Group 3 and Group 4. The next diversification event would have separated the remaining two groups, and these two groups were sister groups from each other. Among these six groups, the Group 6 was the biggest one with high support value, which contained GST members mainly from *Taenia, Echinococcus, Hydatigera* and *Mesocestoides*. For the *S. mansoni*, its members were scattered in Group 2, Group 3 and Group 4, and with most members concentrated in the Group 4.

## Discussion

Our study is the first to report the identification and characterization of GSTs based on all available omic data of *S. mansoni*. We identified 17 new SmGSTs. A search of the NCBI database showed that 15 members belong to cytoplasmic GST and 2 belong to the MAPEG. When classifying the SmGST family members, it was found that the cytoplasmic GST of the family includes Sigma classes besides the common Mu class, which found only in higher eukaryotes, but not in plants, insects and bacteria (36). Our phylogenetic analysis led us to organize the SmGSTs into 2 mian subfamilies. Further analysis revealed that the motif patterns within each group have similar organizations. In *S. mansoni*, most of the SmGSTs displayed ubiquitous but highly variable expression in all tissues/organs studied, which suggested functional divergence. A total of 15 SmGSTs were detected expression in both plerocercoid and adult stages, it demonstrates that members of this gene family are widely distributed and expressed. Bioinformatics analysis revealed SmGST is a hydrophilic protein in consistent with the view that the protein can detoxificate through hydrophilic binding of various exogenous/endogenous toxic molecules and converting them into water-soluble compounds (37).

In addition, one of selected SmGST was successfully expressed in an *E. coli* expression system, and the resulting rSmGST and immune serum were used to define some characteristics of the native SmGST. After purification, rSmGST protein has a good immunogenicity in mice and can be used as an immunogen to produce antibodies. Our results showed that BALB/c mice immunized with the purified rSmGST produced strong specific antibodies to rSmGST. In the IFA analysis, positive staining was widely found in the epidermis and parenchyma of plerocercoid, and in the epidermis, parenchyma, uterus and egg shell of adult worm. qPCR results showed that SmGST gene was expressed in eggs, plerocercoid and adult stage, and the highest expression level was found in adult stage, indicating that GST was related to the development of *S. mansoni*. As described previously, GSTs are known to clear intracellular ROS and assist in redox balance regulation (38). The specific activity of rSmGST toward 1-chloro-2,4-dinitrobenzene was 3.12 μmol min^−1^ mg^−1^, which is comparable to those described for EgGST1 (28), EmGST1 (39) and SGSTM1 (40), indicating that the high activity of rSmGST might play a key role in protecting *S. mansoni* cells against oxidative stress. Differences in optimum pH and temperature for GST activity were observed in various isomers from different species (41). The optimum temperature for rSmGST was 25°C, which was similar to that of GSTs in *Teladorsagia circumcincta, Haemonchus contortus* and *Ancylostoma caninum* (10, 42), lower than the *Echinococcus granulosus* GST (30°C) (16). rSmGST was stable when the temperature ranged from 35 to 50°C, once the temperature exceeded 50°C, the enzyme activity dropped sharply, which was consistent whit previous study (43). SmGST showed the optimal activity at pH 6.5, which was similar to that of *Taenia solium* GST (pH 6.6) (30). Double-reciprocal plots showed uncompetitive inhibition for RB and CB and non-competitive inhibition for the TPT and BSP inhibitors employed. The true inhibition constants, obtained by replotting the slopes from the primary plot vs. CDNB, were in accordance with the inhibition capacity for each inhibitor.

In order to define the relatedness of the proteins across the species, a phylogenetic analysis was performed using GSTs from cestodes. As observed, the cestodes GSTs show complicated phylogenetic patterns, indicating a high level of diversity. Although the GST sequences of *S. mansoni* scattered over multi clades, most of members concentrated in a single group, suggesting that SmGSTs contained both conserved family members and several members in the process of further diversification.

## Conclusion

In this study, firstly, a total of 17 new GST members were identified in *S. mansoni* and an overview of the 17 GST gene expression profiles in different developmental stages were provided. Then, we successfully cloned and expressed the SmGST recombinant protein and studied its enzymatic characteristics. The protein was immunolocalized in the epidermis and parenchyma of plerocercoid, and in the epidermis, parenchyma, uterus and egg shell of adult worm. Purified rSmGST showed high activity at pH 6.5 and optimum temperature at 25°C. The GSH revealed high enzyme affinity to rSmGST, and TPT displayed as a powerful enzyme inhibitor. Finally, the phylogenetic analysis showed a high level of diversity of cestodes GSTs. The SmGSTs contained both conserved family members and several members in the process of further diversification. This study will lay a foundation for further studies on the biological function of GSTs in *S. mansoni* as well as other taxa in which GSTs occur.

## Data availability statement

The datasets presented in this study can be found in online repositories. The names of the repository/repositories and accession number(s) can be found in the article/Supplementary material.

## Author contributions

XZ designed this study. WQC, SSL, CC, and JC performed the experiments. XZ analyzed the data with the assistance of WQC. XZ, WQC, and ZQW wrote the manuscript. All authors have read and approved the final manuscript.

## Funding

This work was supported by National Natural Science Foundation of China (81971956 and U1704189) and Natural Science Foundation of Henan Province of China (212300410070).

## Conflict of interest

The authors declare that the research was conducted in the absence of any commercial or financial relationships that could be construed as a potential conflict of interest.

## Publisher's note

All claims expressed in this article are solely those of the authors and do not necessarily represent those of their affiliated organizations, or those of the publisher, the editors and the reviewers. Any product that may be evaluated in this article, or claim that may be made by its manufacturer, is not guaranteed or endorsed by the publisher.
