# Supplementary material for: Molecular characteristics of glutathione transferase gene family in a neglect medical Spirometra tapeworm
Source: Front Vet Sci. 2022 Nov 2;9:1035767. doi: 10.3389/fvets.2022.1035767 (PMC9666886; doi:10.3389/fvets.2022.1035767)
Supplement: Supplementary file 1 [file Data_Sheet_1.pdf]

Supplementary materials

Table S1. Primers used in qRT-PCR analysis.

Table S2. Putative motifs of SmGSTs.

Table S3. Summary of cestodes glutathione transferases.

Fig S1. Prediction of basic physical and chemical properties of SmGST. (a) Transmembrane prediction; (b) Hydrophobicity prediction; (c) Functional domain prediction; (d) Signal peptide prediction; (e) Subcellular localization prediction; (f) Secondary structure prediction; (g) 3D structure prediction; (h) Phosphorylation site prediction.

Fig S2. (a) Molecular autodock between GST and GSH. Red represents ligands, blue represents acceptor residues, and yellow dashed lines represent hydrogen bonds. (b) Molecular autodock between GST and CDNB.

Fig S3. Immunofluorescence localization of GST in different developmental stages of *Spirometra mansoni*.

Fig S4. Plot composition of fractional velocities versus log [I] for the inhibition of SmGST with bromosulfophthalein (BSP) (rectangles), Cibacron Blue (CB) (blue triangles), Rose Bengal (RB) (circles), triphenyltin chloride (TPT) (green triangles).

Table S1. Primers used in qRT-PCR analysis.

Gene ID	Primer name	Sequence (5'-3')	Product size (bp)
ON527155	ON527155-S<br>ON527155-A	CGGCAGCAAGAAGT<br>AACACGACGGCTCA	98
ON527156	ON527156-S<br>ON527156-A	CCGCCCACTTTCTC<br>CGTTCCTCGGCAGT	170
ON527157	ON527157-S<br>ON527157-A	TCGCTGTCTTACTCGG<br>AGTCGGCGAGTTCC	186
ON527158	ON527158-S<br>ON527158-A	TAAACCGAAGATGCC<br>TGCCAGGAGACGAG	106
ON527159	ON527159-S<br>ON527159-A	GCTTCTGCCACCTT<br>GCACGACACGGATAG	116
ON527160	ON527160-S<br>ON527160-A	GCACAGCCTATCCG<br>CAAGCGTGTATTTCATT	106
ON527161	ON527161-S<br>ON527161-A	TGGTCGTGTTTGCT<br>AAGTCGGCATAAGTG	141
ON527162	ON527162-S<br>ON527162-A	CTGTGGCGAAGAGG<br>AATCAGCATCGTCCT	107
ON527163	ON527163-S<br>ON527163-A	GAAACGGCCAAGCCACTGA<br>CGGGCACTTCCTCCAACATC	136
ON527164	ON527164-S<br>ON527164-A	TGTGATTCTCCGTTAC<br>ATCCGACTTAGACCAG	120
ON527165	ON527165-S<br>ON527165-A	TACCGACCAAAGGA<br>TGCCAGGAGACGAG	145
ON527166	ON527166-S<br>ON527166-A	GTATGTGGCGAGGAG<br>TGCGTCGGTTGTGA	90
ON527167	ON527167-S<br>ON527167-A	GCCATCCGTGTCGT<br>ATGGCAAGGTTTCG	124
ON527168	ON527168-S<br>ON527168-A	CCAAGCCATCCGTCTC<br>CGGCAGGTTAGGAAAGT	142
ON527169	ON527169-S<br>ON527169-A	TGGTCGTGTTTGCT<br>TGAACCACCTCTTACT	113
ON527170	ON527170-S<br>ON527170-A	ATCCGTCTTCTGCT<br>CATCGCCGTCAATA	139
ON527171	ON527171-S<br>ON527171-A	ATTTGGGCGATAAACTCTGGC<br>TCCAAAGCCTCAAATCGTTCC	154
GAPDH	GAPDH-S<br>GAPDH-A	AGCAACCTCGTTGATGTCGT<br>TGAATTGACCGTGGGTGGAG	97

Table S2. Putative motifs of SmGSTs.

Motif	Consensus sequence	Length	No. of GST proteins	e-value
1	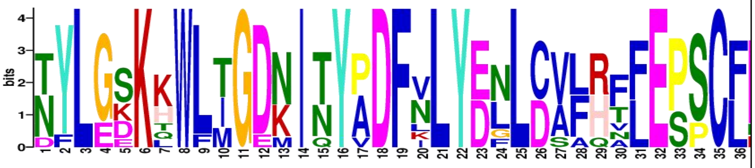 NYLGSKKWLTGDNITYPDFVLYENLCVLHFFEPSCFDKFPNLKEYIERFE	50	9 (Thioredoxin-like or GST_C)	2.6e-215
2	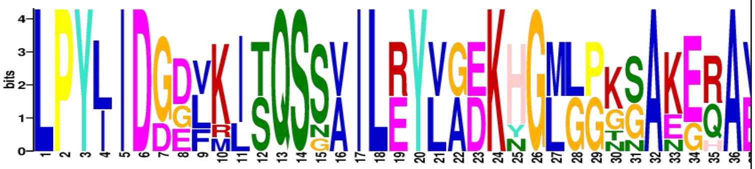 LPYJIDGDLKISQSSVILEYLGEKHGLGPKSAKERAEJAMLZAEJKDLRI	50	8 (Thioredoxin-like or GST_C)	1.6e-161
3	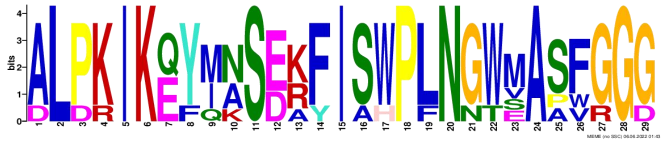 ALPKIKZYMNSEKFISWPLNGWMASFGGG	29	6 (Thioredoxin-like and GST_C)	2.0e-063
4	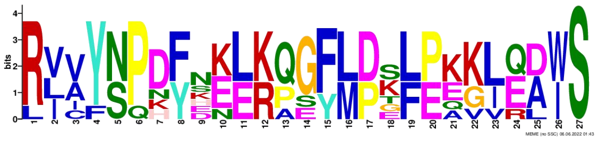 RVVYNPDFNELKQGFLDKLPKKLZAWS	27	7 (Thioredoxin-like and GST_C)	1.4e-033
5	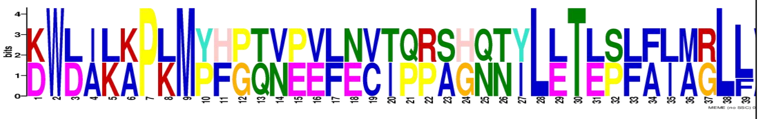 DWDAKKPKMPHPTNPEFEVTPPAGNTYLLTLPFFJAGLLVG	41	4 (2 GST_sigma, 2 MAPEG)	3.7e-026
6	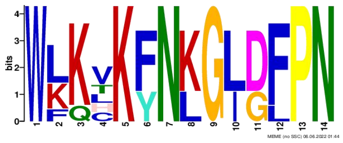 WLKVKFNKGLDFPN	14	6 (Thioredoxin-like and GST_C)	6.5e-023
7	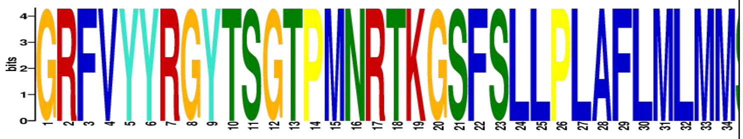 GRFVYYRGYTSGTPMNRTKGSFSLLPLAFLMLMMSVTGVQHLVASIR	47	2 (MAPEG)	1.6e-019

8	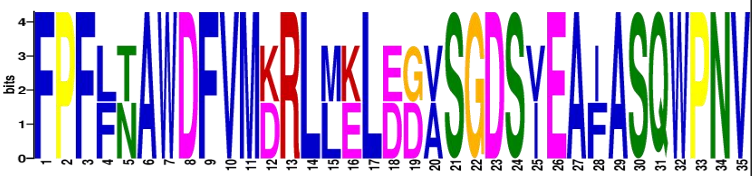 FPFFNAWDFVMDRLLELEGVSGDSIEAFASQWPNV QEYCQLMNQKPFIM	49	2 (GST_C)	2.3e-011
9	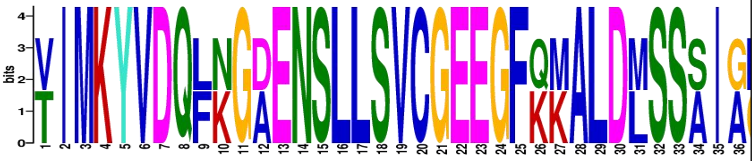 VIMKYVDQFKGDENSLLSVCGEEGFKKALDLSSAI GGPRAKICFSSDATK	50	2 (GST_C)	2.3e-008

Table S3. Summary of cestodes glutathione transferases.

Species	SS	Sequence ID
Acanthocheilonema viteae	5	nAv.1.0.1.g03730, nAv.1.0.1.g01436, nAv.1.0.1.g06673, nAv.1.0.1.g02376, nAv.1.0.1.g06859
Mesocestoides corti	20	MCU_013327, MCU_013154, MCU_003508, MCU_013983, MCU_006477, MCU_008298, MCU_011818, MCU_008297, MCU_011567RA, MCU_011567RB, MCU_011994, MCU_012232RA, MCU_012232RB, MCU_012232RC, MCU_012273, MCU_012923, MCU_013234, MCU_013318, MCU_014131, MCU_014450
Echinococcus canadensis	7	EcG7_07679, EcG7_05160, EcG7_02172, EcG7_00970, EcG7_01502, EcG7_07682, EcG7_06223
Echinococcus granulosus	9	EgrG_000535200, EgrG_000685900, EgrG_000538000, EgrG_000886800, EgrG_000535300, EgrG_000538700, EgrG_000538900, EgrG_000537600, EgrG_000459050,
Echinococcus multilocularis	14	EmuJ_000537600, EmuJ_002163100, EmuJ_000538000, EmuJ_000535200, EmuJ_000538300, EmuJ_000535300, EmuJ_000685900, EmuJ_000538700, EmuJ_000538600, EmuJ_000538500, EmuJ_000538900, EmuJ_000005400, EmuJ_000886800, EmuJ_000459050
Taenia solium	11	TsM_000670100, TsM_000472800, TsM_000610200, TsM_000741700, TsM_000157300, TsM_001229600, TsM_000126000, TsM_000639300, TsM_000610300, TsM_001037400, TsM_000815300
Taenia saginata	12	TSAs00009g02345, TSAs00009g02333, TSAs00041g05719, TSAs00009g02337, TSAs00009g02341, TSAs00003g00983, TSAs00009g02334, TSAs00004g01377, TSAs00009g02332, TSAs00045g06062, TSAs00009g02338, TSAs00009g02343
Taenia asiatica	14	TASK_0001023401, TASK_0001024601, TASK_0000859901, TASK_0000537001, TASK_0000859801, TASK_0000976601, TASK_0000759801, TASK_0000990801, TASK_0000935901, TASK_0000395601, TASK_0000308701, TASK_0000308901, TASK_0000527801, TASK_0000760001
Taenia multiceps	13	Tm7G012789, Tm4G009912, Tm4G009956, Tm4G009913, Tm4G009952, Tm1G001741, Tm1G000278, Tm4G009954, Tm4G009949, Tm4G009955, Tm4G009957, Tm4G009944, Tm4G009947
Hydatigera taeniaeformis	6	TTAC_0000454701, TTAC_0000776701, TTAC_0000995301, TTAC_0000712301, TTAC_0000198001, TTAC_0000712501
Hymenolepis microstoma	15	HmN_000811800, HmN_000614200, HmN_000391300, HmN_000391200, HmN_000295100, HmN_000366200, HmN_003039730, HmN_000366000, HmN_000366500, HmN_000876900, HmN_003039670, HmN_003039680,

		HmN_003039700, HmN_003039710, HmN_003039720
Hymenolepis nana	9	HNAJ_0000263201, HNAJ_0001218801, HNAJ_0001156901, HNAJ_0000005601, HNAJ_0000364501, HNAJ_0000962901, HNAJ_0000304501, HNAJ_0001218901, HNAJ_0000546901
Hymenolepis diminuta	16	HDID_0001100801, HDID_0001107001, HDID_0001120001, HDID_0001006501, HDID_0000663601, HDID_0000662601, HDID_0000656301, HDID_0001006301, HDID_0001120901, HDID_0000646401, HDID_0000536401, HDID_0000665501, HDID_0000035201, HDID_0000573801, HDID_0000989101, HDID_0000230201
Schistocephalus solidus	6	SSLN_0001969301, SSLN_0001455401, SSLN_0001638001, SSLN_0001683301, SSLN_0001208501, SSLN_0000541201
Dibothriocephalus latus	7	DILT_0000100601, DILT_0001646401, DILT_0000130401, DILT_0000521001, DILT_0001366701, DILT_0001237601, DILT_0001846901
Spirometra mansoni	18	ON527155, ON527156, ON527157, ON527158, ON527159, ON527160, ON527161, ON527162, ON527163, ON527164, ON527165, ON527166, ON527167, ON527168, ON527169, ON527170, ON527171, AEI16476.1

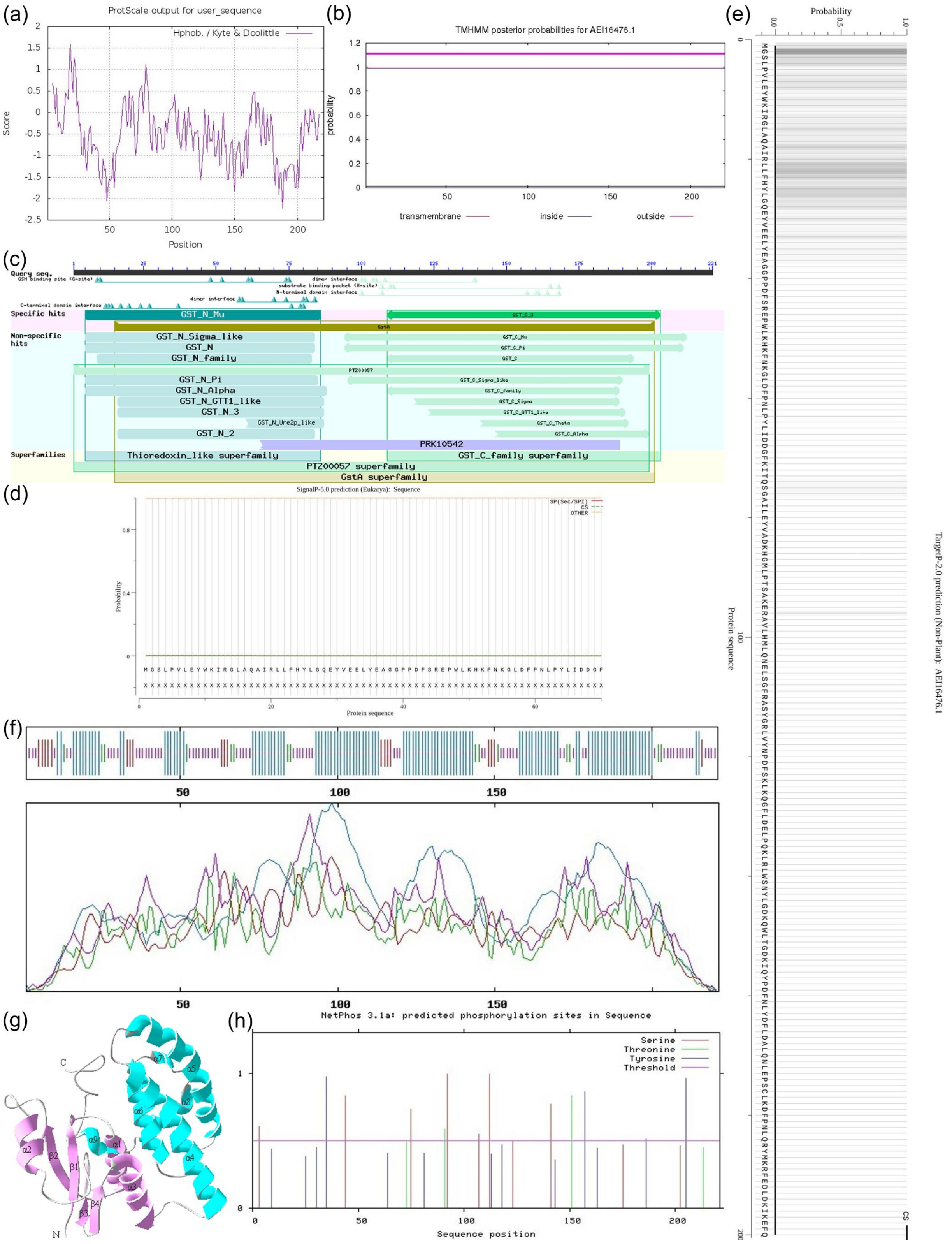

Fig S1. Prediction of basic physical and chemical properties of SmGST. (a) Transmembrane prediction; (b) Hydrophobicity prediction; (c) Functional domain prediction; (d) Signal peptide prediction; (e) Subcellular localization prediction; (f) Secondary structure prediction; (g) 3D structure prediction; (h) Phosphorylation site prediction.

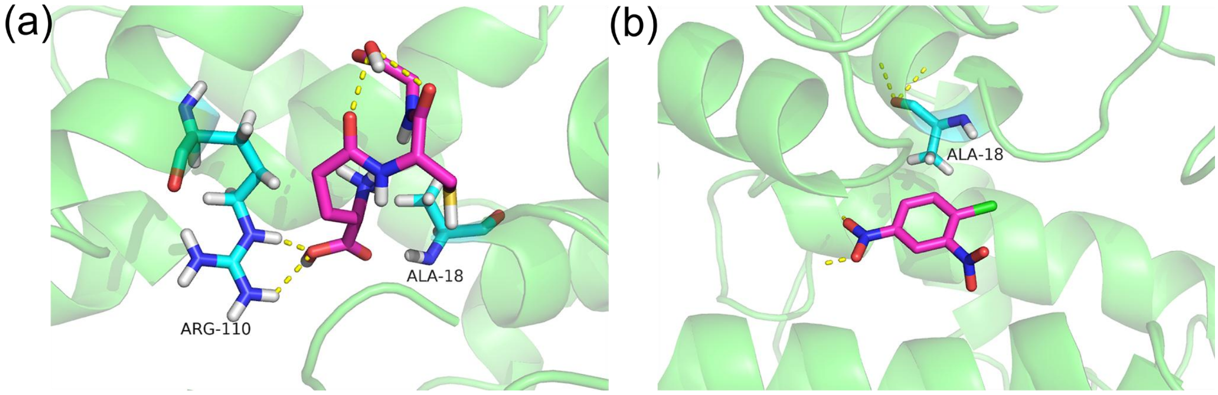

Fig S2. (a) Molecular autodock between GST and GSH. Red represents ligands, blue represents acceptor residues, and yellow dashed lines represent hydrogen bonds. (b) Molecular autodock between GST and CDNB.

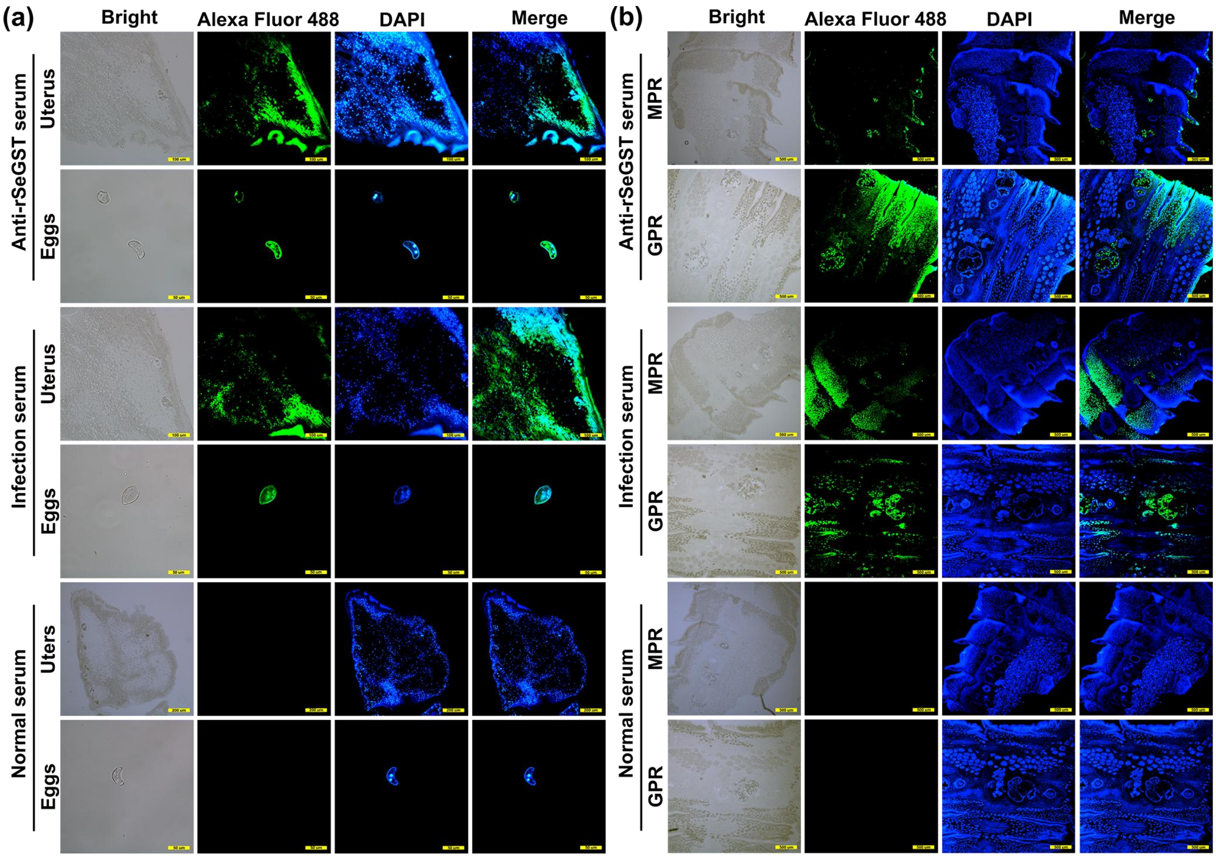

Fig. S3. Immunofluorescence localization of GST in different developmental stages of *Spirometra mansoni*. Green fluorescence is the location of GST protein. Scale of different segments of adult: 500 μm; Uterine: 100 μm; Eggs: 50 μm.

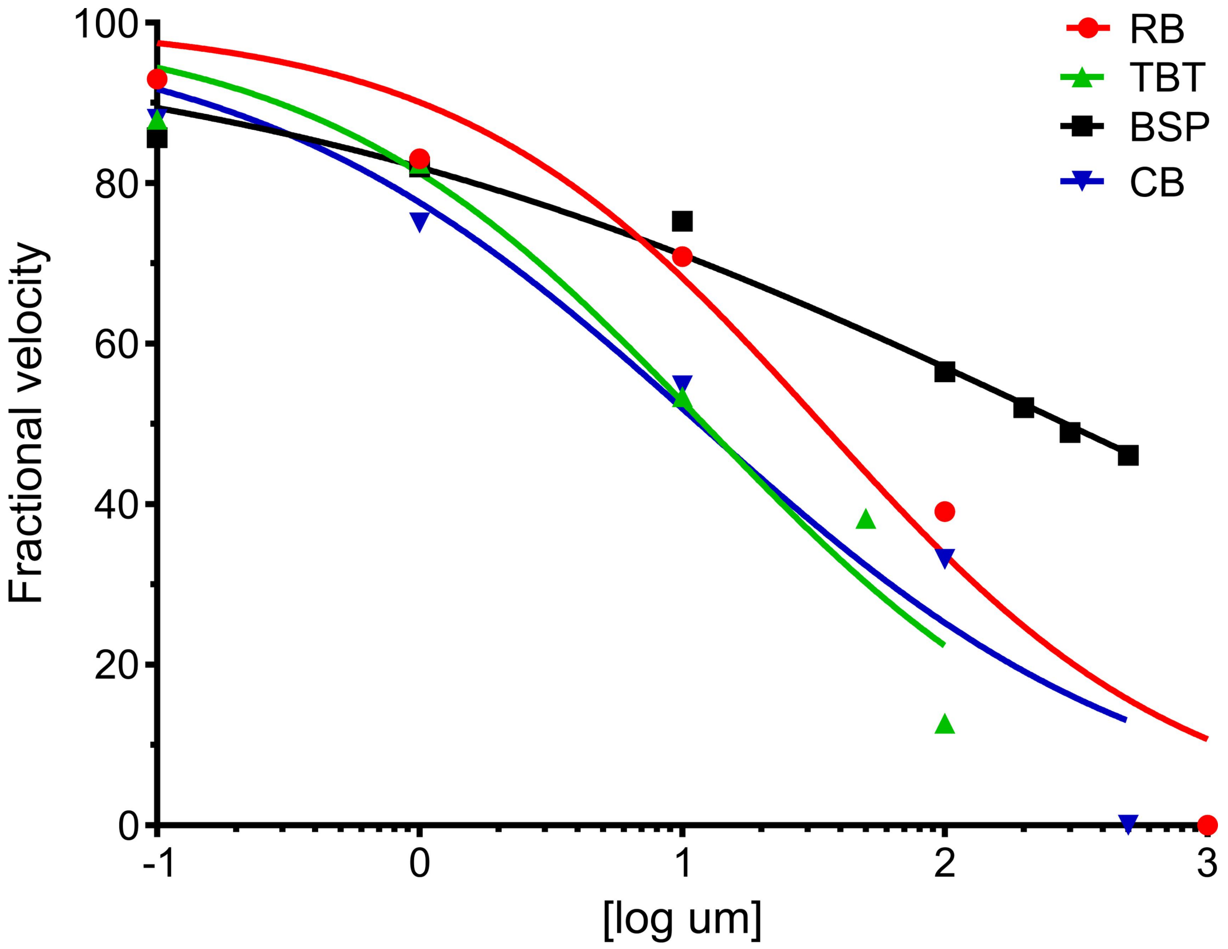

Fig. S4. Plot composition of fractional velocities versus log [I] for the inhibition of SmGST with bromosulfophthalein (BSP) (rectangles), Cibacron Blue (CB) (blue triangles), Rose Bengal (RB) (circles), triphenyltin chloride (TPT) (green triangles).
